# Supplementary figures and images for: Chemokines Kill Bacteria by Binding Anionic Phospholipids without Triggering Antimicrobial Resistance
Source: bioRxiv. 2024 Jul 25:2024.07.25.604863. Preprint. [Version 1] doi: 10.1101/2024.07.25.604863 (PMC11291121; doi:10.1101/2024.07.25.604863)

Figure S1

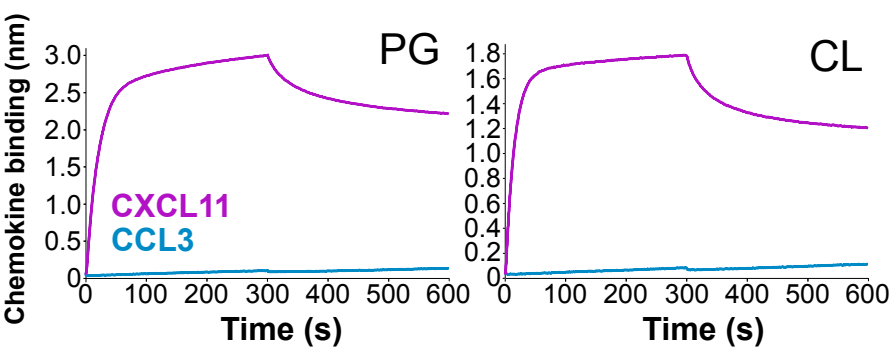

Supplement: Supplement 1 — Figure S1. CCL3 does not bind phosphatidylglycerol or cardiolipin. The binding of CCL3 (500 nM) to phosphatidylcholine liposomes containing 30% PG or CL, as indicated on the top right corner of each graph, was analyzed by BLI. CXCL11 was used as positive control. PG, phosphatidylglycerol; CL, cardiolipin. [file media-1.pdf]

Figure S2

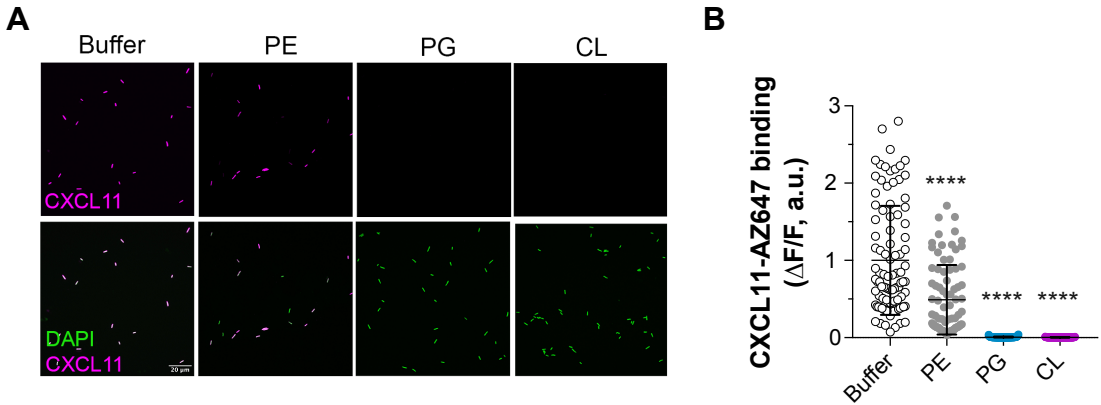

Supplement: Supplement 2 — Figure S2. Liposomes containing PG or CL block CXCL11 binding to bacteria. a) Representative images of the binding of fluorescent CXCL11-AZ647 to bacteria in the presence of buffer or PC liposomes (100 μM) containing 30% of PE, PG, or CL, as indicated above each micrograph column. Top row, images for the staining of CXCL11-AZ647 alone; Bottom row, merge of CXCL11-AZ647 staining with DAPI. A white scale bar (20 μm) is inserted in the bottom left image. b) Quantification of the fluorescence intensity of the binding of CXCL11-AZ647 per bacterium in each treatment group. Each dot represents one bacterium (n ≈ 100). Data are shown as mean ± SD from one experiment representative of 2 independent experiments and were compared to the buffer-treated group using one-way ANOVA with Tukey test for multiple comparisons (****, p<0.0001). DAPI, 4′,6-diamidino-2-phenylindole; CL, cardiolipin; PG, phosphatidylglycerol; PE, phosphatidylethanolamine. [file media-2.pdf]

Figure S3

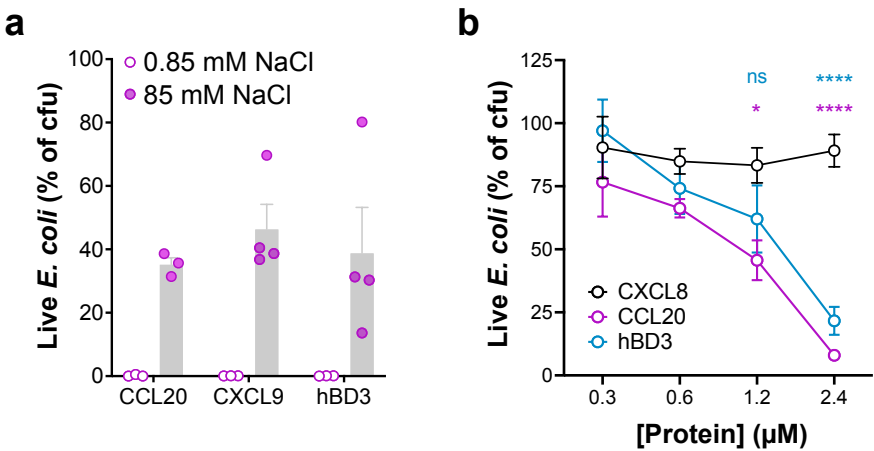

Supplement: Supplement 3 — Figure S3. Chemokines and defensins exert reduced but significant antimicrobial activity at 85 mM NaCl. a) High salt concentrations reduce antimicrobial activity by chemokines. Dot-plot showing a direct comparison of the antimicrobial effect of CCL20, CXCL9 and hBD3 in buffers containing 0.85 mM (open dots) or 85 mM (filled dots) NaCl. Bacteria were incubated with the indicated proteins (1.2 μM) and bacterial viability was analyzed as in a. Each dot corresponds to the mean of one independent experiment analyzed in triplicates. Gray bars indicate the mean ± SEM. b) Chemokines are as potent antimicrobials as hBD3 in the presence of 85 mM NaCl. E. coli (1 × 10+5 cfu) survival after incubation with increased doses of CCL20, hBD3 or CXCL8 (non-antimicrobial control) as indicated in the inset. Lines indicate the % of cfu counted for each sample relative to the cfu counted after treatment of bacteria with buffer alone. Data are summarized as the mean ± SEM of 3 independent experiments analyzed in triplicates. Statistical differences with the CXCL8 control group were analyzed by two-way ANOVA. Results of this statistical analysis (ns, not significant; *, p < 0.05; ****, p < 0.0001) are color-coded and indicated above each concentration data point. hBD3, human beta-defensin 3; cfu, colony forming units; NaCl, sodium chloride. [file media-3.pdf]

Figure S4

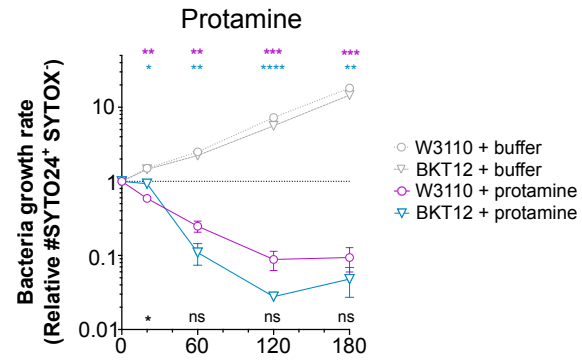

Supplement: Supplement 4 — Figure S4. Protamine is a potent bactericidal peptide. Time-to-kill assay showing the number of live W3110 or BKT12 bacteria over time relative to the initial number of live bacteria (time 0) after incubation with buffer alone or 1.2 μM of protamine (as indicated in the legend). Live bacteria were quantified at different time points by co-staining with SYTO24 (stains dead and live bacteria) and SYTOX (stains only dead bacteria) using FACS. Horizontal dotted line indicates the ratio = 1 (no growth). Data are summarized as the mean ± SEM of 3 independent experiments, each performed with 3 biological replicates. Data were analyzed by two-way ANOVA with Bonferroni test for multiple comparisons (ns, not significant; *, p < 0.05; **, p < 0.01; ***, p < 0.001; ****, p < 0.0001). Results of statistical analyses for the comparison of buffer vs protamine for each bacterial strain are color-coded and indicated above each graph. Results of statistical analyses for the comparison of protamine-treated W3110 vs BKT12 are indicated above the x axis. [file media-4.pdf]

Figure S5

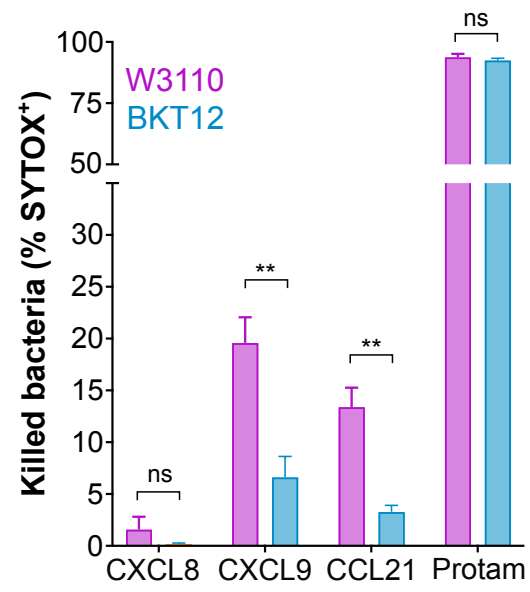

Supplement: Supplement 5 — Figure S5. CL-deficient bacteria are resistant to direct killing by antimicrobial chemokines. Quantification of the % of dead W3110 and CL-deficient BKT12 bacteria (as indicated in the inset) 90 min after treatment with buffer alone or 4.8 μM of CXCL9, CCL21 or protamine. Dead bacteria were quantified by FACS as the % of SYTOX+ cells relative to the total number of SYTO24+ bacteria. Data are the mean ± SD of 3 biological replicates from one experiment representative of 3 independent experiments. Statistical differences (W3110 vs BKT12) were analyzed by two-way ANOVA with Bonferroni test for multiple comparisons (ns, not significant; **, p < 0.01). Protam, protamine. [file media-5.pdf]

Figure S6

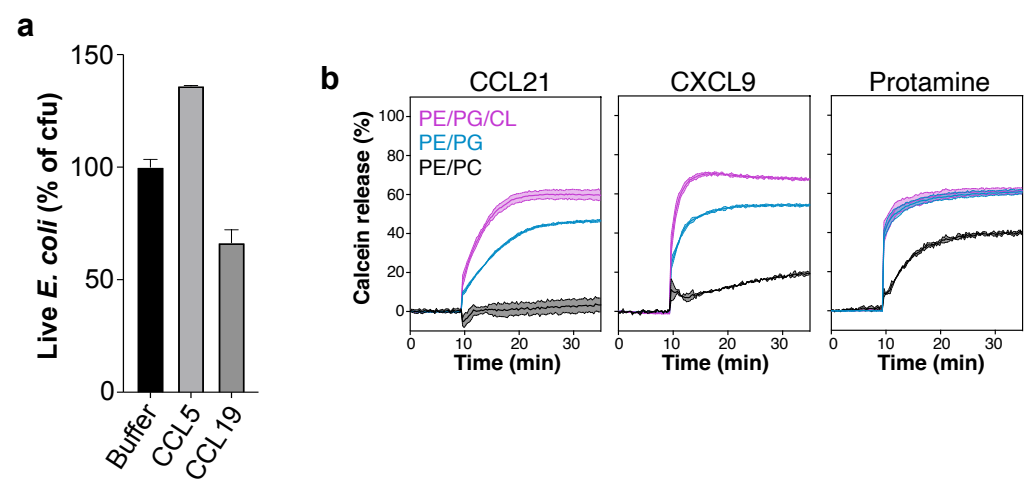

Supplement: Supplement 6 — Figure S6. CCL5 is not antimicrobial and CL promotes liposomal membrane disruption by low doses of antimicrobial chemokines. a) E. coli (1 × 10+5 cfu) survival after 2 h incubation with buffer alone or 1.2 μM of CCL5 or CCL19 as indicated on the x-axis. Bars represent the mean ± SD of cfu counted for each treatment on TSB-agar plates. b) Calcein-leakage assays showing the % of calcein released by 0.15 μM of CCL21, CXCL9 or protamine (as indicated above each graph) from PE/PG/CL, PE/PG or PE/PC liposomes (CCL21 graph inset) relative to the maximum calcein release observed when liposomes were incubated with TritonX-100. Solid lines represent the mean of 3 biological replicates. Colored shaded area around the lines represents the SD. Data are from one experiment representative of 3 independent experiments. cfu, colony forming units; CL, cardiolipin; PG, phosphatidylglycerol; PE, phosphatidylethanolamine; PC, phosphatidylcholine. [file media-6.pdf]

Figure S7

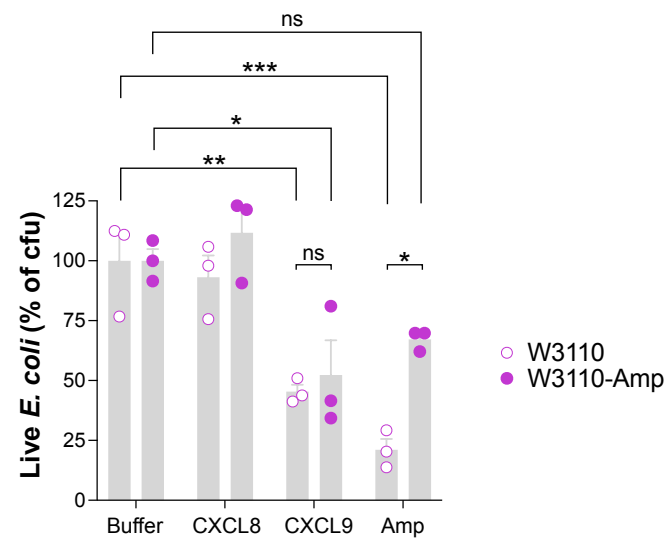

Supplement: Supplement 7 — Figure S7. CXCL9 kills ampicillin-resistant E. coli. Parental W3110 and ampicillin-resistant strain W3110-Amp (as indicated in the legend) were incubated with buffer, 1.2 μM of CXCL8 (non-antimicrobial) or CXCL9 (antimicrobial), or 10 μg/ml of ampicillin (Amp) for 2h at 37°C. Bacterial viability was determined by cfu assays. Bars represent the mean ± SEM % of surviving bacteria relative to the “Buffer” group for each bacterial strain. Data are summarized from 3 independent experiments performed in triplicate and were analyzed by two-way ANOVA with Tukey’s test for multiple comparisons (ns, not significant; *, p < 0.05; **, p < 0.01; ***, p < 0.001). cfu, colony forming units. [file media-7.pdf]
